# Underinvestment in diagnostic and vaccine research & development has put the world at greater risk from the Bundibugyo virus epidemic in 2026

**DOI:** 10.1080/21505594.2026.2710544

**Published:** 2026-08-09

**Authors:** Hinh Ly

**Affiliations:** Department of Veterinary & Biomedical Sciences, College of Veterinary Medicine, University of Minnesota, Twin Cities, MN, USA

On 16 May 2026, the World Health Organization (WHO) declared the current outbreak of Bundibugyo Ebola virus (BDBV) in the Democratic Republic of Congo (DRC) and Uganda a public health emergency (https://www.who.int/emergencies/alert-and-response). BDBV (*Orthoebolavirus bundibugyoense*) is a lesser-known member of the genus *Orthoebolavirus*. The other Ebola virus species known to cause severe and deadly hemorrhagic fever disease in humans include *Zaire ebolavirus* (*Orthoebolavirus zairense*, ZEBOV), Sudan virus (*Orthoebolavirus sudanese*, SUDV), and Taï Forest virus (*Orthoebolavirus taiense*, TAFV). BDBV was named after the Bundibugyo District of western Uganda, where the first known outbreak occurred in August 2007, followed by a second outbreak in the DRC in August 2012 [1,2] The 2026 Ebola outbreak in the DRC and Uganda is the third known BDBV outbreak. Whereas the reported case fatality rates for ZEBOV infection are very high (60–90%), those of the BDBV are lower (34–46.8%) [3]. Yet, BDBV is highly pathogenic and capable of inflicting severe disease and sustained human-to-human transmissions, as demonstrated in the current and previous outbreaks [1].

While the Ervebo®, a recombinant vesicular stomatitis virus-vectored Ebola virus (rVSV-ZEBOV) vaccine, has been successfully developed and tested against ZEBOV, its effectiveness against BDBV is unknown [4]. As a result, the WHO has recommended that Ervebo® and other experimental Ebola vaccines be evaluated through appropriately designed clinical trials during the ongoing outbreak. Although Ervebo® was developed for ZEBOV, recent immunogenicity evaluation indicates that it may elicit detectable cross-reactive antibody responses against the BDBV glycoprotein, albeit at substantially lower levels than those elicited by the ZEBOV glycoprotein antigens. As such, the degree to which this vaccine can confer clinical protection against BDBV infection remains unknown [5]. As a result, the Coalition for Epidemic Preparedness Innovations (CEPI) has stepped up its effort of supporting the development and testing of multiple experimental EBOV vaccines, which include an rVSV-based vaccine developed by the International AIDS Vaccine Initiative (IAVI), a chimpanzee adenovirus ChAdOx1-vectored EBOV vaccine developed by the University of Oxford and manufactured by the Serum Institute of India, and an mRNA-based EBOV candidate developed by Moderna [6].

The current BDBV outbreak has revealed a major vulnerability in EBOV outbreak preparedness. According to preliminary findings from the WHO, delays in laboratory confirmation tests, as diagnostic tests were initially focusing on the dominant ZEBOV and less on other EBOV species, had led to undetected community transmission in eastern DRC [7]. Other factors also play important roles in the current outbreak, including the ongoing regional armed conflict, population displacement, relatively porous borders between the DRC and its neighboring countries, and weak healthcare infrastructure. These – and perhaps other underappreciated – factors complicate the planning and implementation of outbreak control strategies, such as isolation, contact tracing, infection prevention, and safe burial practices (https://www.who.int/emergencies/alert-and-response). Notably, a relatively high rate of infections among healthcare workersx during the early stages of the outbreak highlights a shortfall in infection prevention and control.

The ongoing BDBV outbreak, as of July 27th, 2026, has accounted for over 3,300 confirmed cases, 1,489 deaths, and another 321 suspected cases in the DRC, and 20 confirmed cases, 2 deaths, and 1 probable death in the neighboring country, Uganda (Table 1) (https://www.who.int/emergencies/alert-and-response). Another imported case to France has also been reported as of June 24^th^, 2026 (https://www.cdc.gov/ebola/situation-summary/index.html). The US Centers for Disease Control and Prevention (CDC) has modeled scenario projections for the current BDBV outbreak and has recommended large-scale, rapid public health action to control the current outbreak before it becomes one of the largest EBOV epidemics in history (https://www.cdc.gov/mmwr/volumes/75/wr/mm7522e1.htm). Data extracted from the Institut National de Sante Publique (INSP) Situation Reports (SitReps) from May 14^th^ to June 18^th^, 2026 showed an exponential growth pattern of infections typical of the early epidemic phases (Figure 1(A)), with the steepest increases in the number of daily new cases occurring in late May through early June of 2026 (Figure 1(B)). Although EBOV is less transmissible than SARS-CoV-2, increased human travel and migration can facilitate its rapid geographical spread. Other factors, such as deforestation, mining, human population displacement due to military or civil unrest, climate and/or ecosystem changes, can unintentionally facilitate the emergence of zoonotic transmissions of EBOV and other hemorrhagic fever-causing viruses in Africa, such as LASV [8–10].

**Figure 1.**
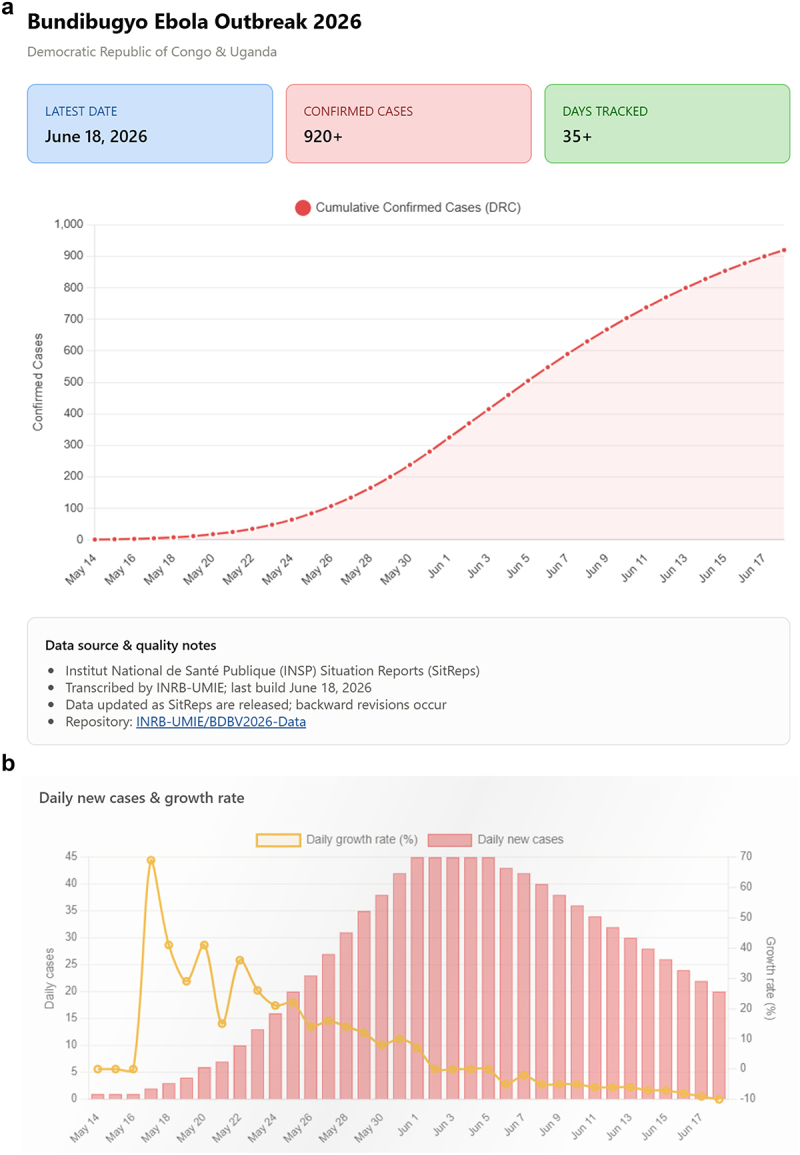
Epidemiological curve of the cumulative confirmed case counts over time in the DRC and Uganda. The cumulative confirmed case counts reached 920+ cases by June 18th, 2026, which were tracked across 12 reporting health zones in the DRC, with additional cases detected in Uganda through imported infections. Data from the DRC were from the GenomicEpi.com (https://www.genomicepi.com/outbreaks/ebola-2026/), which aggregated data from the Institut National de Sante Publique (INSP) situation reports (SitReps) (https://insp.Cd/category/sitrep/) and deposited at INRB-UMIE/BDBV2026-data website, whereas data from Uganda were from the Uganda Ministry of Health dashboard (https://evd-daily.Health.go.ug/). Data extracted from SitReps from May 14th to June 18th, 2026 showed an exponential growth pattern of infections typical of the early epidemic phases (A), with the steepest increases in the number of daily new cases occurring in late May through early June of 2026 (B).

**Table 1.** Daily update (as of July 2nd–3rd, 2026) of the number of BDBV cases in the DRC and Uganda as reported by the WHO. Table and data are derived and adapted from WHO website: https://www.who.int/emergencies/alert-and-response.

Affected countries	Suspected cases	Confirmed cases	Confirmed deaths	Probable cases	Probable deaths	CFR (%) confirmed	Recovered
**Data as of 27 July 2026**							
Democratic Republic of the Congo	321	3,360	1,487	–	–	44%	597
**Data as of 21 July 2026**							
Uganda	–	20	2	1	1	10%	18
**Data as of 6 July 2026**							
France	–	1	0	0	0	–	1
**Total**	321	3,381	1,489	1	1	44%	616

There is a renewed call for adopting a broader, virus species-inclusive approach in diagnostic assay development as well as in developing universal EBOV vaccine(s) and other immuno-therapeutics, such as broadly neutralizing monoclonal antibodies that are capable of eliciting protection against multiple EBOV species [11,12]. For example, it has been shown that a formulation of two broadly neutralizing monoclonal antibodies (MBP134^AF^) can provide protection against multiple EBOV species (ZEBOV, SUDV, and BDBV) in a preclinical ferret model, highlighting its potential as a universal EBOV immune-therapeutic modality [13] that needs to be evaluated through appropriately designed clinical trials during the ongoing outbreak. Toward this end, sustained global interest and investment combined with accelerated human clinical trials are necessary to translate new EBOV vaccines, antivirals, and other immuno-therapeutic approaches into timely outbreak prevention and response. Conventional public health interventions and supportive care are equally important and have led to 597 recovered patients in the DRC among 3,360 confirmed cases and 18 out of 20 recovered individuals in Uganda. These approaches alone have significantly reduced the EBOV case fatality rate from 60–90% in some past EBOV outbreaks to about 44% in the DRC and to just 10% in Uganda during the current BDBV outbreak (https://www.who.int/emergencies/alert-and-response).

The recent outbreak has also emphasized the need for better approaches to genomic surveillance in endemic regions. New, affordable, agile technologies, such as the Oxford Nanopore MiniION sequencing and/or other multiplex diagnostic platforms, can help quickly identify the culprit before it causes widespread infection. It is noteworthy that in the current outbreak, several weeks elapsed before BDBV was definitively identified, which allowed for unchecked BDBV transmissions during the early phase of the epidemic (https://www.who.int/emergencies/alert-and-response). In summary, a comprehensive response to BDBV and other emerging zoonotic pathogens requires a concerted global effort in research and development that includes but is not limited to vaccine, antiviral, and immuno-therapeutic development, coordinated and strengthened surveillance system with locally capable and accessible diagnostic facilities, sustained international support for local healthcare system and workers in Africa (and other underdeveloped countries), public health education, and appreciation for the One Health approach that addresses the important interactions between human, animal, and their environments.

## Data Availability

No primary data is included in the article.

## References

[1] MacNeil A, Farnon EC, Wamala J, et al. Proportion of deaths and clinical features in Bundibugyo Ebola virus infection, Uganda. Emerg Infect Dis. 2010;16(12):1969–4. doi: 10.3201/eid1612.100627 21122234 PMC3294552

[2] Wamala JF, Lukwago L, Malimbo M, et al. Ebola hemorrhagic fever associated with novel virus strain, Uganda, 2007–2008. Emerg Infect Dis. 2010;16(7):1087–1092. doi: 10.3201/eid1607.091525 20587179 PMC3321896

[3] Hulseberg CE, Kumar R, Di Paola N, et al. Molecular analysis of the 2012 Bundibugyo virus disease outbreak. Cell Rep Med. 2021;2(8):100351. doi: 10.1016/j.xcrm.2021.100351 34467242 PMC8385243

[4] Kuehn R, Ryan H, Okwaraeke KC, et al. Vaccines for preventing Ebola virus disease. Cochrane Database Syst Rev. 2024;11(11):Cd015828. doi: 10.1002/14651858.Cd015828 39560047 PMC11574942

[5] Lhomme E, Wiedemann A, Ayouba A, et al. Cross-reactive Bundibugyo antibody responses after licensed Ebola vaccines. medRxiv. 2026. doi: 10.64898/2026.05.27.26354223 42485649

[6] Baraniuk C. Ebola: three vaccines rushed into development for Bundibugyo outbreak. BMJ. 2026;393(e557985):e557985. doi: 10.1136/bmj-2026-557985 42229937

[7] Matson MJ, Chertow DS, Munster VJ. Delayed recognition of Ebola virus disease is associated with longer and larger outbreaks. Emerg Microbes Infect. 2020;9(1):291–301. doi: 10.1080/22221751.2020.1722036 32013784 PMC7034085

[8] Hudu SA, Jimoh AO, Amin-Nordin S, et al. Environmental degradation as a recipe for emerging viral diseases: implications for global health. One Health Outlook. 2026;8(1). doi: 10.1186/s42522-026-00203-w PMC13003655 41851794

[9] Brisse M, Ly H. Hemorrhagic-fever-causing arenaviruses: lethal pathogen and potent immune suppressor. Front Immunol. 2019;10:372. doi: 10.3389/fimmu.2019.00372 30918506 PMC6424867

[10] Murphy HL, Ly H. Pathogenicity and virulence mechanisms of lassa virus and its animal modeling, diagnostic, prophylactic, and therapeutic developments. Virulence. 2026;12(1):2989–3014. doi: 10.1080/21505594.2021.2000290 PMC8923068 34747339

[11] Holtsberg FW, Shulenin S, Vu H, et al. Pan-ebolavirus and pan-filovirus mouse monoclonal antibodies: protection against Ebola and Sudan viruses. J Virol. 2016;90(1):266–278. doi: 10.1128/jvi.02171-15 26468533 PMC4702560

[12] Russo L, Villani L, Ieraci R, et al. Global Health preparedness frameworks and recombinant vaccine platforms: a public Health perspective on regulations and System readiness. Vaccines (Basel). 2026;14(2):144. doi: 10.3390/vaccines14020144 41746065 PMC12945048

[13] Bornholdt ZA, Herbert AS, Mire CE, et al. A two-antibody pan-ebolavirus cocktail confers broad therapeutic protection in ferrets and nonhuman Primates. Cell Host Microbe. 2019;25(1):49–58.e45. doi: 10.1016/j.chom.2018.12.005 30629918 PMC6341996

